# A Practitioner’s Guide to Performing a Holistic Evaluation of Technology-Enhanced Learning in Medical Education

**DOI:** 10.1007/s40670-019-00781-7

**Published:** 2019-08-02

**Authors:** James D. Pickering, Michelle D. Lazarus, Jennifer L. Hallam

**Affiliations:** 1 Division of Anatomy, Leeds Institute of Medical Education, School of Medicine, University of Leeds, Worsley Building, Clarendon Way, Leeds, West Yorkshire LS2 9NL UK; 2 Centre for Human Anatomy Education, Department of Anatomy and Developmental Biology, Monash University, Melbourne, Victoria Australia; 3 Leeds Institute of Medical Education, School of Medicine, University of Leeds, Leeds, West Yorkshire UK

**Keywords:** Technology-enhanced learning, Evaluation, Medical education

## Abstract

Technology-enhanced learning (TEL) is now a common mode of educational delivery within medical education. Despite this upsurge, there remains a paucity in comprehensive evaluation of TEL efficacy. In order to make meaningful and evidence-informed decisions on ‘how’ and ‘when’ to utilise technology within a course, ‘useful knowledge’ is required to support faculty in these decision-making processes. In this monograph, a series of pragmatic and achievable approaches for conducting a holistic evaluation of a TEL resource intervention are detailed. These suggestions are based on an established TEL evaluation framework, as well as the author’s own experience and that of the broader literature. The approaches cover development of an appropriate research question that is based on the availability of existing TEL resources alongside the peer-reviewed literature; the development of an appropriate team as well as recommendations for navigating ethical approval; conducting small-scale quantitative and qualitative measure; and performing a large-scale mixed methods assessment to understand the holistic impact of the TEL resource.

## Introduction

Medical education has seen a rapid upsurge in the use of technology to support and enhance the delivery of core curriculum components. This change in approach is observed across a number of disciplines within medical education, such as the teaching of basic and clinical science content [1, 2], and clinical skills education (e.g., including point-of-care ultrasound [3]), to support the collection of student data in the workplace and thereby supporting timely and meaningful workplace-based assessment feedback [4]. Although this innovative approach to medical education has grown, often referred to as technology-enhanced learning (TEL) in the literature, numerous authors draw attention to the perceived lack of robust evidence to justify its inclusion, specifically highlighting a paucity of meaningful evidence to support colleagues in integrating such technology into their curricula [1, 5]. Recently, this perception has been supported by evidence within anatomy education that highlights the majority of TEL evaluations are based primarily on student satisfaction alone, with a shortfall in methodologies to collect robust empirical data documented [6]. Against this backdrop of changing educational delivery and practice, and with the growing call for increased research and evaluation into its efficacy [7–9], this monograph details pragmatic and achievable approaches to conduct a holistic evaluation using a recognised TEL evaluation model (TELEM) that consists of four levels [10]. Within this context, the overarching aim of this monograph is to provide practitioner-based colleagues with pragmatic, tangible, and achievable methodologies and evaluation principles. Examples from the TEL literature, where available, are provided and signposted throughout. The TEL principles can be applied as part of an evaluation process prior to implementation, as part of a curriculum review, or as a basis for a research project across the general theme of scholarship of teaching and learning.

## Summary of the Technology-Enhanced Learning Evaluation Model

**Fig. 1:**
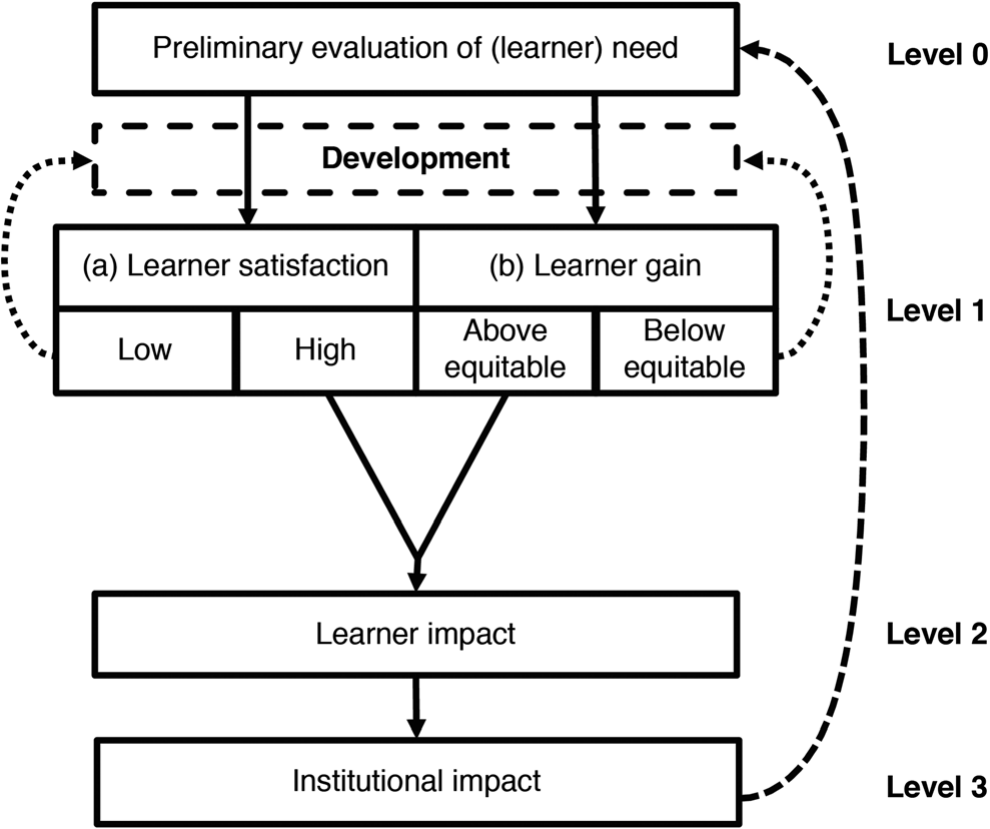
The technology-enhanced learning evaluation model (TELEM) used as a basis for the methodologies and principles outlined across 4 levels: Level 0 (Evaluation of Learner Need), Level 1a (Learner Satisfaction), Level 1b (Learning Gain), Level 2 (Learner Impact), and Level 3 (Institutional Impact; modified from Pickering and Joynes, 2015(10))

The TELEM provides a clear and comprehensive framework for practitioners to use when evaluating the impact of a TEL resource within medical education [10]. It consists of four levels that can be deployed individually or consecutively depending on the evaluation rationale and resources available within the host institution (Fig. 1). A detailed explanation of the TELEM is available in the original published article, with a concise summary provided below:

*Level 0* is a needs analysis that assesses the requirements and rationale for introducing a TEL resource into a teaching session or wider programme of education. This stage of the evaluation process aims to ensure that technology is the appropriate solution to either a curriculum problem or is an alternative approach to delivering the specific learning objectives of the programme.

*Level 1* focuses on learner satisfaction with the new tool (Level 1a—learner satisfaction) and the impact on learning within a controlled environment (Level 1b—learning gain). Level 1a attempts to understand the degree to which users (i.e., students) are generally satisfied with the technological resource, with the main goal of this level being to better understand if it is user-friendly and enjoyable. Level 1b seeks to explore the underlying impact on learning gain (i.e., changes in knowledge retention) within a controlled environment, typically, via a randomised controlled trial (RCT) approach.

*Level 2* takes a broader view to assess the impact of the TEL resource on users within an active curriculum and takes into consideration all the other available learning opportunities. This approach, therefore, aims to draw out the specific impact such an intervention has on student outcomes alongside other resources and, in combination with data from *Levels 1* and *2*, provides a holistic view of the impact a specific TEL resource has on student learning.

*Level 3* draws on the work by Walsh et al. [11] and is the most complex level, requiring input from students and numerous stakeholders to make judgements on the institutional benchmarks used to evaluate the overall success of the intervention in terms of ‘value for money’. Due to the detailed financial methodologies used to calculate the impact of such an intervention, specific details are outside the scope of this manuscript. Simply, each level of the TELEM is aligned with a return on investment strategy, resulting in Level 1a being associated with a cost-utility analysis, Level 1b with a cost-effectiveness analysis, Level 2 with a cost-benefit analysis, and an evaluation that delivers across all levels (Levels 0–3) is associated with a full cost-feasibility analysis.

**Fig. 2:**
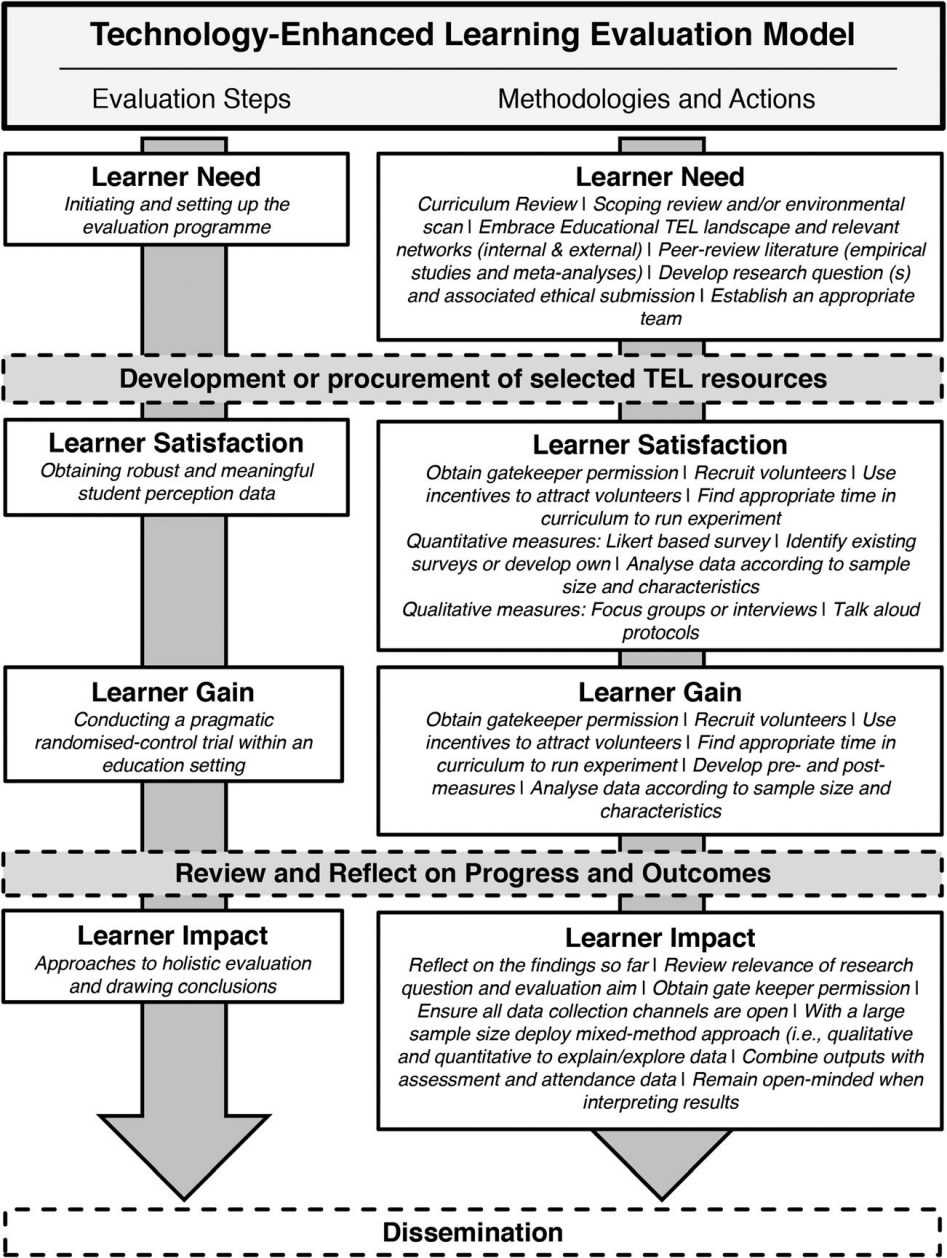
Flow diagram linking Levels 0–2 of the technology-enhanced learning evaluation model to pragmatic and achievable methodologies. TEL, technology-enhanced learning

The monograph is divided into four sections that provide recommendations on the following: (1) initiating and setting up the evaluation programme (Level 0—Learner Need); (2) obtaining robust and meaningful student perception data (Level 1a—Learner Satisfaction); (3) conducting a pragmatic randomised controlled trial within an education setting (Level 1b—Learner Gain); and (4) drawing conclusions and approaches to holistic evaluation (Level 2—Learner Impact; Fig. 2). Each recommendation is formed from the experience of the authors and the wider literature where appropriate. Although many of the methodologies and principles detailed can be used across higher education disciplines more broadly, given the well-documented rise in technological approaches to curriculum delivery within medical education, it would seem reasonable to reaffirm such practical guidance, especially for practitioners who do not have a grounding in education research within the context of TEL.

## Initiating and Setting up the Evaluation Programme (Learner Need)

Prior to embarking on a new project, it is important to initially set the research outcomes that you aim to achieve. To support this process, a pragmatic and reflective approach is often helpful to draw on, and learn from, previous experiences as either a teacher or educational researcher. This level of self-reflection is particularly important for practitioners who want to embark on educational research within their own practice, and establishing realistic expectations from the onset will help to maintain focus and perspective [12]. In developing and trying to deliver a project of this nature, a considerable amount of time, planning, and effort will be required. It is advisable to find an experienced mentor to provide honest support and set expectations, especially as educational research can be particularly messy and reveal ambiguous results [13]. Although frameworks and practical solutions will help to provide answers and complete the ‘jigsaw’, an acceptance that some questions will remain unanswered is a mindset to appreciate early on. With educational research, there is often no silver bullet [14].

Alongside the need to position yourself within the appropriate context and establishing a network to provide ongoing support and reassurance, an understanding and awareness of the wider educational technology sector is required. Although it might not be necessary at this stage to comprehensively explore the peer-reviewed literature and get bogged down in detail, obtaining a clear understanding of the general area and tools available that may be applicable to your situation and setting is vital (i.e., a general needs assessment). To support the development of this knowledge base, an initial scoping or environmental scan of the grey literature (i.e., materials, resources, or research outputs that are documented or published outside of the traditional distribution services such as academic or commercial publishers) would be advisable by searching and engaging with non-academic sources, such as teaching resource publishers, online repositories, and industrial partners as these are particularly relevant in the ever-changing TEL context (e.g., NMC Horizon Report on innovations in tertiary education is available here: Becker et al., (2017) [15] and Hatch and Pearson (2005) provide a methodology for conducting an environmental scan [16]). Although seeking advice and support from external sources is valuable and provides an alternative view of what is possible, connecting with local networks can also be a rich source of information. Connecting with colleagues with similar problems or intentions, but from other disciplines, may generate ideas and open up the possibility for multi- or cross-disciplinary collaborations (i.e., a targeted needs assessment).

After the successful scoping of the literature and having established the specific area of the curriculum that would benefit from a TEL-based enhancement, engaging with the relevant peer-reviewed literature will now be essential as you begin to formulate your research questions and integrate the appropriate methodologies into your teaching context [17, 18]. It is important at this stage to make sure you are not ‘re-inventing the wheel’ and conducting work that has already been completed, albeit in other institutions, but under similar conditions. One difficulty with educational research is the generalisability of the findings beyond your own institution’s educational context [19]. However, a way around this issue is to source reliable and relevant meta-analyses that investigate similar areas and can help you to gain a picture of what the current research-base indicates and whether, in fact, there is a sufficient evidence in existence to guide your TEL resource deployment or develop your research question. Having undertaken this thorough examination of the available literature, an evidence-informed research question, which is grounded in the relevant literature and serves the need of your context, will be formed [20]. An important consideration, when formulating your research question and the methodology used to try and answer it, is to consider the impact this intervention will have on the student cohort. With practitioner-based research that utilises the student cohort within the respective programme, it is essential that the methodologies employed do not hinder the successful delivery of the course and negatively affect student education. These considerations need to be explained in detail during the ethical submission process and are explored in more detail in the following section.

Once the research questions and desired outcomes of the research are established, it is important to determine what expertise will be required to support its successful delivery. The project lead should take ownership and be confident that each member of the team holds the necessary skills, or can develop the knowledge base, to successfully discharge their role. As well as skills and expertise, behavioural traits of team members are also important. For example, some may be excellent communicators or networkers and can effectively bring the team together, whereas others may be skilled in looking at the ‘bigger picture’ and how the research adds to the wider literature or to specific learning outcomes. Furthermore, diversity of team members is key in order to bring different perspectives and ideas to assimilate learning and inform each stage of research from design to dissemination [21]. Depending on the research design, specific roles may be required such as statisticians to conduct quantitative analysis, qualitative researchers to run focus groups or interviews and lead on the synthesis of this data, and curriculum experts who will be able to advise on the implementation of the intervention. Try to avoid getting enveloped into the mantra of ‘the bigger the problem, the bigger the team’, but draw on the expertise and skill sets of those people who will ‘buy-in’ to the project and can provide the necessary support. Frank discussions early in the project about commitment, existing workload, and authorship of any subsequent research outputs are important so everyone is clear of their expectations. It is increasingly recognised that cross-discipline team working is often a catalyst for innovation [22], and therefore calling on colleagues from neighbouring departments, such as Schools of Education and developed educational research centres, will be beneficial and provide different perspectives and ideas that both support the delivery and constructively critique the methodology. This aspect of the project delivery team is particularly important if the solution to your problem is technology based. With the rapidly changing educational technology environment, having a team member who is familiar with this area will help scope potential solutions.

As with any research project that utilises human participants, an ethics application to the relevant ethical review board is necessary. The formulation of this application is therefore an essential piece of the research project and can be used to help plan, refine and clarify your research project’s methodologies [23]. The assessment panel will review the risks and benefits for participants by evaluating your methodology and ensure the project is fair, worthwhile and deliverable in the time available. Whilst you may be able to evaluate your TEL intervention without ethical approval for the purposes of curricular reform, submitting an application is a worthwhile activity to ensure clarity of purpose and it is essential if you are planning on sharing the results of your project outside of the research team (e.g., educational scholarship networks, conference presentations, manuscript submission). The ethical review board will also assess if the project is equitable for all students within the cohort and that suitable access to the TEL intervention is put in place, so everyone receives the same learning experience. It would be deemed unfair if only a proportion of students receive the intervention, and cross-over methodologies are an appropriate approach to try and remedy this issue [24]. Considerations to accessibility and digital literacy are to be specifically focused on here; although it is perceived that certain generations of students are inherently adept at using technology to enhance their education, this is not always the case [25–27]. In addition, whilst some institutions employ a ‘bring your own device’ policy, it is important to ensure that if a TEL intervention is chosen, all students receive the same levels of access and have the necessary training on how to use the resource most effectively [28]. Furthermore, the ethical review board will assess the possibility of coercion across the project and steps should be in place by the research team to prevent this from taking place—it is highly unlikely that approval for your project would be granted without these assurances. As a result of this planning and in-depth consideration of impact on the student cohort, educational research projects are often front-loaded, that is, considerable work is undertaken initially to ensure all the necessary data is captured as the educational programme is underway. It is important, therefore, to plan early and troubleshoot potential problems that may occur during the study [29].

Within the ethical application for your evaluation, you will need to have detailed the specific methodologies that you intend to use to answer the research question. The following sections provide examples that can be used either as individual approaches, or as a joined-up detailed evaluation, to investigate the efficacy of the specific TEL resource. These methodologies focus on student feedback for the usability and appeal of the resource, its impact on learner gain in a controlled environment, and a holistic understanding of the resource within an active programme. These approaches are aligned with TELEM at levels 1a, 1b, and 2 (Fig. 1).

## Obtaining Robust and Meaningful Student Perception Data (Learner Satisfaction)

The focus of TELEM at Level 1a is to obtain a greater understanding on student perceptions of the resources and typically entail engaging in a conversation with the end user. A common and relatively straight forward approach to obtaining this data is through quantitative enquiry that focusses on gathering new information that is related to the research question and is grounded in the positivist paradigm [30]. The principle involves gathering attitudes, behaviours, and opinions from your target sample to generate objective information. Questionnaires, predominantly using Likert scales, are a popular research tool within social sciences research and adopt a deductive reasoning approach where questions or statements (items) are pre-designed to gather specific information. Often, there will be existing surveys that have been designed and rigorously validated to assess specific areas of interest or social constructs. Therefore, the most appropriate place to start would be scoping the peer-reviewed literature for existing scales that are specific to the concepts of your research question. However, there may not always be a scale that is fit for your specific purpose and you may have to develop a new measure [31]. The principles and psychology of survey design and measurement are pivotal when creating valid and reliable questionnaires. Consideration of elements such as cognitive load and question structure, alongside the wider principles of validity and generalisability, is important to maintain methodological rigour and ensure that the conclusions are not misleading [32]. What you decide to do with the results from this survey will largely depend on the size and representative nature of the sample population; with a wide range of parametric and non-parametric approaches available, choosing the most appropriate approach will be based on how you decide to treat the data. Typically, descriptive data from small samples will be reported as percentages, means and standard deviations, or median and interquartile range, respectively. Given the exploratory nature of Level 1a in TELEM, it is highly likely that the sample size will be small. With a larger data set, more sophisticated and inferential approaches may be used, and this is likely to be available once feedback is collected from the whole cohort who had access to the resource once it was integrated. This area of statistical analysis is considered further below.

An additional approach to obtaining user feedback is through qualitative measures. Qualitative methodology is a popular and well-established approach to collecting student perceptions of the intervention. This methodology sometimes relies on data collected through open-ended questions via focus groups or interviews and allows the researcher to delve deeper into the actual experiences that are not measurable by quantitative approaches [33]. Using the cohort of students who have received the TEL intervention at this preliminary stage, you should ask for volunteers to inquire about participants’ opinions, view, and thoughts on the new resource. Depending on what feedback you require from the intervention will largely determine how and when the focus groups are conducted. For example, if you want a group to work together and discuss the TEL resource intervention and its impact, then focus groups with 4–6 participants would be an appropriate choice. However, if questions are likely to provoke some personal, emotional, or sensitive responses, structured- or semi-structured interviews, where the participant will likely feel less social pressure to answer in a certain way, would be a more appropriate option. To effectively conduct the sessions, the research team should generate questions and prompts from both the wider literature, the quantitative survey data, and a well-structured programme of qualitative enquiry. These prompts are used to facilitate the discussion and ensure you are answering the research question and utilising the time available most effectively. An additional approach to evaluating the usability of technology-based resources is to use a ‘talk aloud’ protocol [34, 35]. This approach can either be performed alongside a facilitator or the participant can have their interaction with the resource video recorded. The main output is the user describes their interaction with the resource which is then transcribed and appropriately coded for analysis.

Although these approaches to evaluation are common across the education research landscape, in the context of TELEM, Level 1a is aimed at acquiring essential information on the resource prior to its full integration into an active curriculum. This tactical investigation into the student perceptions is in contrast to the large-scale feedback that will be possible if the decision is made to integrate the resource across a programme. Alongside an understanding of student perceptions with the new TEL resource, understanding its impact on learning gain with controlled ‘experimental’ conditions is considered at Level 1b, with this methodology utilising RCTs that are common across bioscience research.

## Conducting a Pragmatic Randomised Controlled Trial (Learner Gain)

In order to make a more meaningful decision whether to integrate a TEL resource into a programme, understanding its effectiveness and efficiency in supporting the acquisition of knowledge is essential. One approach to assessing the ability of the TEL resources effectiveness and efficiency—‘learning gain’—is to develop a RCT. Although deemed to be the gold standard in biomedicine and clinical science, the RCT is less utilised in educational research and has received well-grounded criticism [14]. However, notwithstanding these criticisms, it is possible to conduct a RCT that aims to limit the number of confounding variables that may influence the linear cause and effect assumptions made with this approach. In order to conduct a RCT with your TEL resource, you will need to recruit volunteers from a representative cohort. Depending on the timing of your call, you will need to ensure that your recruitment is ethically appropriate: if you are utilising curriculum time, then you will need to ensure all additional resources are available to the entire cohort; if not, then you will still need to make sure that the time taken up with this involvement does not impinge on other timetabled teaching sessions or assessments. These details should have been discussed during the planning of your project and would have been approved by the ethical review board. Typically, RCTs will utilise pre- and post-test measures that seek to explore changes in knowledge, application, or skills, and utilise between- and within-group statistical analyses that include traditional quantitative approaches, such as parametric tests (i.e., *t* tests, analysis of variance) on absolute and normalised gain values conducted [36, 37]. Although this approach is not common in medical education, numerous studies have utilised such an approach in an attempt to limit confounding variables and obtain a statistically robust understanding on effectiveness. Moreover, depending on the level of delay and staggering of the post-test design, an idea of the TEL resource efficiency can also be discerned. A pragmatic and consistent approach would be to consult the existing literature on how these studies have been conducted and adapt these to your research question. For example, in Physics and Cell Biology education Hake, and Knight and Wood, 2005, [38–40] have detailed the use of absolute and normalised gain calculations to measure changes in learning gain, with Colt et al., Issa et al., and Pickering, 2016, [41–43] utilising similar methodologies to assess changes in learning gain after a surgical skill course, a lecture series on shock, and a TEL intervention in anatomy education, respectively. Although this level of evaluation is potentially artificial in nature as it is not assessing the role of the TEL resource within the wider curriculum, it does provide a robust and effective approach to obtain clean and unbiased data that is specifically focused on the impact of the resource.

## Approaches to Holistic Evaluation and Drawing Conclusions (Learner Impact)

Depending on your methods to deploying these approaches, either in isolation during an active curriculum, or in advance of its integration, you will now have a baseline understanding of its impact on the student population in relation to its perceived utility and enjoyment, and the degree to which it can support the acquisition of knowledge in comparison with an existing approach or more traditional resource. With this information available, you can now make an informed decision as to whether (or how) resource deployment within a larger cohort or active curriculum is best executed. If you decide to integrate the resource within an active curriculum, you should continue to evaluate its impact, and with the potential for a greater number of end users, the methodological approaches can be suitably developed and scaled to try and draw out robust and meaningful inferences. As students put considerable faith in the resources proposed by their instructors, it is important that these resources are of an acceptable quality and they should promote enjoyment, alongside efficient and effective learning. However, the decision to implement and integrate a TEL resource into an active cohort is necessarily more nuanced requiring consideration of additional factors. Alongside this research, curriculum experts and course leaders from your team should be consulted on how best this resource can be integrated to ensure the curriculum as a whole is still deliverable.

Having decided to release the TEL resource into an active curriculum, the next stage would be to assess if it is having a meaningful impact on student performance and engagement. This will require clarity on what aspect of the curriculum you are expecting the resource to have an impact on and to make sure all the data collection channels are open and able to collect the relevant information. As detailed previously, this work will need to be front loaded so you are gathering the data from the beginning of the course and you will need to have gatekeeper permission to expose students to a new intervention. An effective way to ensure you are collecting the necessary data is to run a mock analysis that requires you to visualise what you need and what you already have in relation to the research question, and thereby clarifying exactly what you need to collect. Moving from the RCT aspect where it is likely the numbers of volunteers would be manageable due to the preliminary nature of the project, assessing the curriculum impact will inevitably involve a much larger dataset. As this work is focused on TEL resources, utilising the built-in tools from your institution’s learning management system (LMS)-specific datasets can be an efficient and effective way to collect user data. LMS data collected will need to be checked for accuracy and evaluated for the ability to be exported in a format that allows you to manipulate it by a range of fixed variables (i.e., gender). With the recent use of learning analytics [44], this whole process can be streamlined and made easier, but importantly, the researcher still needs to know what to look for and, therefore, what to ask the ‘analytics’ to provide. The interface between the data analyst and the educational researcher is key in providing data that is useable, meaningful, and ethical. This approach to data analytics will allow you to assess the impact the resource has had in comparison with other resources and support any correlational activity to assess the impact on student outcomes. Although this area of educational enquiry may be problematic for a number of reasons, including the reliability in using download statistics as a reliable proxy for meaningful usage, engagement, and learning [45, 46], it should not be discounted, rather it is best to be transparent and consider these findings within the appropriate context of the environment.

Alongside the collation of the LMS large dataset or more sophisticated learner analytic approach, you can also re-run the original or a modified version of the questionnaire that was used previously to obtain a large-scale understanding of student opinion or other affective aspects of their behaviour with the resource. The modified version of the questionnaire should be based on the preliminary work that was used to inform the decision to integrate the resource, with a more robust mixed methods approach utilised to provide a clear and holistic understanding of the TEL resources’ impact. Large-scale mixed methods research is often a longitudinal process using qualitative and quantitative approaches to explore and/or explain the data being gathered, such as assessment results and attendance. Mixed methods research works on the principle of using one research methodology to gather data which is then interpreted to inform the next stage [47]. This can be exploratory, where data from the first stage (quantitative via a Likert-based questionnaire) is explored and a detailed narrative is constructed that informs the second stage (qualitative via focus groups), or explanatory, where rich data from a smaller sample size at stage one (qualitative via focus groups) is explored for generalisability in a larger sample in stage two (quantitative via a Likert-based questionnaire). Mixed methods research can improve the rigour and/or depth of your study whilst also minimising the confounding variables that are inherent in medical education research.

At this stage of the process, you would have received considerable data and spent hours conducting the project and analysing the findings. With this vast collection of primary data, it is important to try and make sense of it all by delving into the detail and drawing out any conclusions. Having an open mind and allowing the results to direct the next stage of research is important and focusing on student impact can help to determine future directions. In terms of evaluating the impact of a TEL resource in student education, being clear about what you determine a ‘success’ is important. Whether this is an improvement in assessment scores, attendance rates, or satisfaction of resource, using a mixed methods evaluative approach will allow you to ascertain if success is due to the resource or if it is explained by additional factors. Being able to explore and explain your data allows you to perform a holistic inquiry and be confident of both your research findings and the impact of your innovation on student education in order to justify a generalisable conclusion. For example, did the TEL intervention provide an added benefit in learning gains or student engagement, or did the outcomes mirror what was already taking place? Did the analysis of the intervention open up additional research questions that were not obvious at the onset? These are important considerations to keep in mind when summarising and making sense of your project, especially as educational research is not simply about seeing simple cause and effect relationships. Having attempted to answer these questions and make sense of the data, this ‘useful knowledge’ can now be used to inform decisions at your own institution and also a global audience by attending conferences and producing research manuscripts [9]. This wider dissemination is becoming increasingly possible, with faculty development workshops, to support colleagues in developing research and evaluation approaches in medical education, appearing alongside traditional research output presentations. Engaging and developing multiple output channels will increase the projects’ impact and help both others and yourself to design and develop new ideas to continually grow the body of evidence to support the decision-making process.

## Conclusions

Education research is sometimes perceived as being messy and/or lacking in conclusive findings that one might expect in clinical or bioscience research. This monograph has attempted to provide educational practitioners and researchers with a number of pragmatic and achievable research methodologies aligned to a TEL evaluation model that provides an opportunity to obtain a holistic view of impact. Specifically, these approaches are described across 4 levels: Learner Need, Learner Satisfaction, Learner Gain, and Learner Impact. In utilising a broad range of methodologies to develop a holistic understanding of impact, informed decisions can be made that allow for sensible and pragmatic changes to educational practice both at the host institution and through considered and timely dissemination across the educational landscape more generally.
